# Nanostructured BaCo_0.4_Fe_0.4_Zr_0.1_Y_0.1_O_3-δ_ Cathodes with Different Microstructural Architectures

**DOI:** 10.3390/nano10061055

**Published:** 2020-05-30

**Authors:** Lucía dos Santos-Gómez, Javier Zamudio-García, José M. Porras-Vázquez, Enrique R. Losilla, David Marrero-López

**Affiliations:** 1 Dpto. de Química Inorgánica, Universidad de Málaga, Cristalografía y Mineralogía, 29071 Málaga, Spain; zamudio@uma.es (J.Z.-G.); josema@uma.es (J.M.P.-V.); r_losilla@uma.es (E.R.L.); 2 Department of Physical and Analytical Chemistry, Oviedo University-CINN, 33006 Oviedo, Spain; 3 Dpto. de Física Aplicada I, Universidad de Málaga, 29071 Málaga, Spain

**Keywords:** BaCo_0.4_Fe_0.4_Zr_0.1_Y_0.1_O_3-δ_, spray-pyrolysis, cathode microstructure, solid oxide fuel cell

## Abstract

Lowering the operating temperature of solid oxide fuel cells (SOFCs) is crucial to make this technology commercially viable. In this context, the electrode efficiency at low temperatures could be greatly enhanced by microstructural design at the nanoscale. This work describes alternative microstructural approaches to improve the electrochemical efficiency of the BaCo_0.4_Fe_0.4_Zr_0.1_Y_0.1_O_3-δ_ (BCFZY) cathode. Different electrodes architectures are prepared in a single step by a cost-effective and scalable spray-pyrolysis deposition method. The microstructure and electrochemical efficiency are compared with those fabricated from ceramic powders and screen-printing technique. A complete structural, morphological and electrochemical characterization of the electrodes is carried out. Reduced values of area specific resistance are achieved for the nanostructured cathodes, i.e., 0.067 Ω·cm^2^ at 600 °C, compared to 0.520 Ω·cm^2^ for the same cathode obtained by screen-printing. An anode supported cell with nanostructured BCFZY cathode generates a peak power density of 1 W·cm^−2^ at 600 °C.

## 1. Introduction

Although solid oxide fuel cells (SOFCs) are considered as one of the most promising technologies for electrical generation, they typically operate at elevated temperatures (700–1000 °C). This results in several problems for commercial implementation, such as high maintenance costs and premature degradation of the cells components. Therefore, decreasing the operating temperature is one of the principal goals to make this technology commercially viable as well as promoting its potential use in non-stationary energy production systems [1,2,3,4].

The efficiency of SOFC at low operating temperatures is mainly limited by the oxygen reduction reactions (ORR) in the cathode [5,6,7,8]. In the last few years, alternative cathode materials to the traditionally used La_0.8_Sr_0.2_MnO_3_ (LSM) have been investigated, such as La_0.6_Sr_0.4_Co_0.2_Fe_0.8_O_3-δ_ (LSFC), Ba_0.5_Sr_0.5_Co_0.8_Fe_0.2_O_3-δ_ (BSCF), GdBaCo_2_O_5+δ_, PrBaCo_2_O_5+δ_ and La_2_NiO_4+δ_ [9,10,11,12,13,14,15]. However, these cathodes have some drawbacks compared to the traditional LSM. For instance, LSCF suffer from superficial phase segregations at high temperatures, which reduces the ORR activity after long-term operation [16,17]. Layered perovskites with high Co-content exhibit high thermal expansion coefficients compared to the electrolytes; and BSCF degrades over time due to phase transformation from cubic to hexagonal structure [18]. 

Recently, BaCo_0.4_Fe_0.4_Zr_0.1_Y_0.1_O_3-δ_ (BCFZY) has been proposed as an efficient cathode material with mixed oxide-ion, proton and electronic conductivity [19,20]. BCFZY was firstly developed as a cathode for proton conductor SOFC; however, it has been recently reported as a favourable material for SOFC, showing attractive chemical and thermal cycling stability [21]. Despite the fruitful findings achieved for this compound, further studies are essential to optimize the microstructure since no investigation has been reported into this field up to now.

The development of new electrodes by microstructural engineering is fundamental to obtaining efficient SOFCs, operating at temperatures below 600 °C. In particular, these should have a stable microstructure, high porosity and surface area, and consequently a long triple phase boundary (TPB) for the ORR [4,22,23]. Diverse strategies have been explored to enhance the cathodic efficiency, including: (i) the infiltration/impregnation method into a porous scaffold, although this is not adequate for mass industrial use due to the multiple fabrication steps required and long time consuming; and (ii) the optimization of the electrode porosity by the use of sacrificial templates, such as polymer and carbon microspheres, or freeze-casting method [24,25]. Alternatively, (iii) the employ of active layers, such as Bi_2_O_3_–based films and mixed conductors, improves the oxygen transfer at the electrode/electrolyte interfaces and reduces the ohmic losses [26,27,28]. 

It is evident that the electrode manufacturing procedures have to be cost-effective, i.e., not time-consuming and easily scalable at an industrial level. Most of the reported preparation methods are tedious and require multiple fabrication steps from the preparation of the electrode powders to deposition and sintering. In this context, spray-pyrolysis deposition has resulted to be an effective method to obtain electrodes over large areas in a single deposition step, reducing considerably the production costs and time. This method has been used to obtain single oxides, such as ZnO and CuO and more complex mixed oxides such as La_0.6_Sr_0.4_Co_0.2_Fe_0.8_O_3-δ_ and PrBaCo_2_O_5+δ_ [29,30,31,32,33]. Unlike the physical methods, such as magnetron sputtering and pulsed laser deposition, the cation stoichiometry of the compounds is easily tailored. In addition, the variation of the deposition conditions allows the obtention of electrode microstructures with different morphologies, from dense thin film to thick porous layers.

In this work, we demonstrate that the electrochemical efficiency of the BaCo_0.4_Fe_0.4_Zr_0.1_Y_0.1_O_3-δ_ cathode is further improved by microstructural architecture design. For this purpose, cost-effective, reproducible and easily scalable preparation methods based on spray-pyrolysis are employed. The structure and morphology have been investigated by X-ray diffraction and electron microscopy. A complete electrochemical study is carried out to understand the electrode mechanism for the ORR as a function of the temperature and the oxygen partial pressure. The results are compared with a traditional cathode obtained from freeze-dried powders and deposited by screen-printing.

## 2. Materials and Methods 

### 2.1. Materials Synthesis

Ce_0.9_Gd_0.1_O_1.95_ (CGO) was used as electrolyte. Commercial powders (Rhodia, Frankfurt, Germany) were pressed into pellets of 0.1 and 1 cm of thickness and diameter, respectively, and sintered at 1400 °C for 4 h. After that, some of the sintered pellets were symmetrically screen-printed with an ink obtained by mixing CGO and Decoflux (Zschimmer and Schwarz, Villarreal, Spain,) in a 50:50 wt. % ratio, and then sintered at 1100 °C for 1 h to obtain a porous backbone of approximately 7 µm thickness. 

For the preparation of the BaCo_0.4_Fe_0.4_Zr_0.1_Y_0.1_O_3-δ_ (BCFZY) cathode by spray-pyrolysis, the stoichiometric amounts of Ba(NO_3_)_2_, Co(NO_3_)_2_·6H_2_O, Fe(NO_3_)_3_·9H_2_O, ZrO(NO_3_)_2_·6H_2_O and Y(NO_3_)_3_·6H_2_O (Merck/Sigma-Aldrich, Barcelona, Spain, purity above 99%) were dissolved in distilled water under continuous stirring. Ethylenediaminetetraacetic acid (EDTA), previously dissolved in diluted NH_3_, was added in a ligand:metal relation of 1:1 and stirred to obtain a dark-purple solution with a cation concentration of 0.02 mol·L^−1^. Notice that a diluted precursor solution is need to avoid a rapid growth of the layers, which results in cracked electrodes. The resulting precursor solution was sprayed onto both faces of the CGO pellet with and without porous backbone layers at 300 °C for 1 h. The spray-pyrolysis deposition conditions were similar to those described in detail elsewhere [29]. After that, the electrodes were treated in a furnace in air at 950 °C for 30 min to achieve crystallization.

For comparison purposes, BCFZY powders were prepared using the freeze-drying method [34]. The same cation solution used in the spray-pyrolysis technique, but with a cation concentration of 0.06 mol·L^−1^, was dropped and fast frozen into liquid nitrogen. The water was gradually sublimated in a Scanvac Coolsafe freeze-dryer for 48 h. The dried powders were put into an oven for two successive thermal treatments, the first one at 300 °C for 1 h to partially decompose the organic material, and the second at 950 °C for 30 min to achieve crystallization. The resulting cathode powders were mixed with Decoflux to obtain an ink, which was screen-printed onto CGO pellets and sintered at 1000 °C for 1 h to ensure appropriate adherence. 

Hereafter, for simplicity reasons, the samples will be labelled as: FD for electrodes obtained from freeze-dried precursor powders, SP for spray-pyrolyzed layers onto polished CGO surface, and SP-CGO for spray-pyrolysis onto porous CGO backbones.

### 2.2. Materials Characterization

X-ray powder diffraction (XRD) were performed by using a PANalytical Empyrean diffractometer (PANalytical, Almelo, The Netherlands) with a CuK_α1,2_ radiation in the 2*θ* range of 10–80° for 1 h. Structural analysis by Rietveld method was carried out with the X’Pert HighScore and GSAS suite software (3.0.5, PANalytical, Almelo, The Netherlands ) [35,36]. 

The morphology and microstructure of the cell components were examined by Field Emission Scanning Electron Microscopy (FE-SEM, Helios Nanolab 650, Hillsboro, OR, USA).

The area specific resistance (ASR) of the symmetrical FD, SP and SP-CGO cells was determined by impedance spectroscopy with a Solartron 1260 impedance/gain-phase analyzer (Hampshire, UK) in the frequency range 0.01−10^6^ Hz. The AC amplitude was varied between 25 and 100 mV, obtaining reproducible results. Pt ink (METALOR^®^ 6082, Metalor, Oullins, France) was painted on both faces of the pellets and fired at 800 °C to obtain a current collector layer. The impedance spectra were recorded as a function of the temperature (400–650 °C) and the oxygen partial pressure (10^−3^–1 atm) in order to study the different processes involved in the ORR [37,38]. The impedance spectra data were fitted by equivalent circuit models with the ZView software (2.9c, Scribner Associates, Southern Pines, NC, USA) [39].

### 2.3. Fuel Cell Evaluation

The performance of BCFZY in real fuel cell operating conditions was evaluated by fabricating Ni-CGO/CGO/SP-CGO anode supported cells. Firstly, a NiO-CGO (60:40 wt. %) composite anode was prepared by mixing the corresponding amount of CGO with an aqueous solution of Ni(NO_3_)_2_·6H_2_O (Merck/Sigma-Aldrich 99%). The water was slowly evaporated by heating under constant stirring; the resulting mixture was heated in an oven at 800 °C for 1 h to decompose the nitrates into NiO. Glassy carbon microspheres (15 vol. %) were added to create an adequate porosity. The anode composite was compacted and pre-sintered at 1000 °C for 1 h, obtaining a 13 mm diameter pellet. Secondly, the electrolyte was made by screen-printing a CGO ink onto the NiO-CGO pellet and then cosintered at 1400 °C for 4 h to densify the electrolyte. A porous CGO backbone layer was screen-printed and sintered onto the electrolyte. Finally, BCFZY was deposited by spray-pyrolysis through a shadow mask of 0.25 cm^2^ and calcined at 950 °C for 30 min.

The NiO-CGO/CGO/SP-CGO cell was mounted in a homemade electrochemical cell and sealed with Ceramabond 668 ceramic paste (Aremco, Northbrook, IL, USA). The current-voltage and impedance curves were acquired with a potentiostat/galvanostat/FRA (Bio-Logic VSP) in the temperature range of 500–600 °C, while air and humidified H_2_ (3 vol. % H_2_O) were continuously supplied to the cathode and anode, respectively.

## 3. Results and Discussion

### 3.1. Structural Analysis

**Figure 1:**
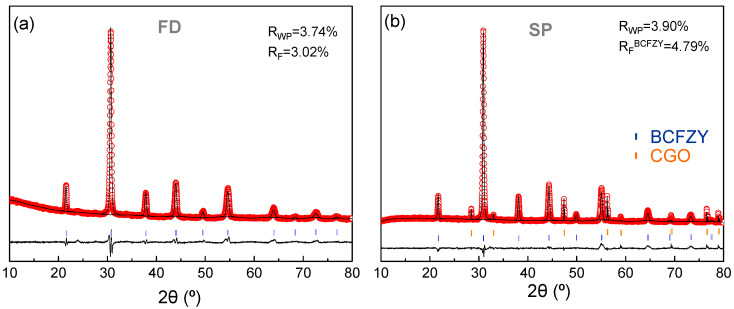
X-ray powder diffraction (XRD) Rietveld fitting of BaCo_0.4_Fe_0.4_Zr_0.1_Y_0.1_O_3-δ_ (BCFZY) cathodes prepared by (**a**) freeze-drying method and (**b**) spray-pyrolysis onto Ce_0.9_Gd_0.1_O_1.95_ (CGO) substrate.

XRD patterns of BCFZY powders and layers prepared from freeze-dried precursors (FD) and spray-pyrolysis onto CGO substrates (SP), respectively, are compared in Figure 1. Both samples are single phases after calcining at 950 °C, showing comparable average crystal sizes, between 30 and 40 nm, which were calculated by the Scherrer equation after correction for instrumental broadening. The similar crystal size is attributed to the identical thermal treatment conditions of FD powders and SP layers. However, the FD electrode suffers a significant grain growth up to 100 nm after screen-printing and sintering at 1000 °C for 1 h, needed to achieve enough adherence with the electrolyte. Thus, SP samples exhibit lower crystal size due to fewer preparation steps and lower sintering temperature, reducing the cation diffusion and the particle growth. 

Notice that the XRD data of SP shows two well-differentiated crystalline phases attributed to BCFZY and the CGO substrate (Figure 1b). No secondary phases are observed, which demonstrate a good chemical compatibility between BCFZY and CGO up to the studied temperature, preventing any performance losses.

The Rietveld method was employed to analyze the XRD data. A cubic perovskite type structure (s.g. *Pm-3m*) and a cubic fluorite (s.g. *Fm-3m*) were considered for the refinements of BCFZY and CGO, respectively. Occupancy factors were adjusted to the corresponding stoichiometries. Background, scale factor, unit cell, isotropic displacement and peak shape parameters were refined, obtaining low disagreement factors of R_wp_ = 3.74 and 3.90%; and R_F_ = 3.02 and 4.79% for FD and SP samples, respectively (Figure 1). 

Regarding the unit cell parameters, these are similar for both preparation methods ~4.082 Å, which is very close to those reported by other authors ~4.08 Å, indicating that a material with the same structure is obtained [21]. This value is somewhat higher than that reported for isostructural Ba_0.5_Sr_0.5_Co_0.8_Fe_0.2_O_3-δ_ (BSCF) ~3.98 Å [40].

### 3.2. Morphology of the Electrodes

**Figure 2:**
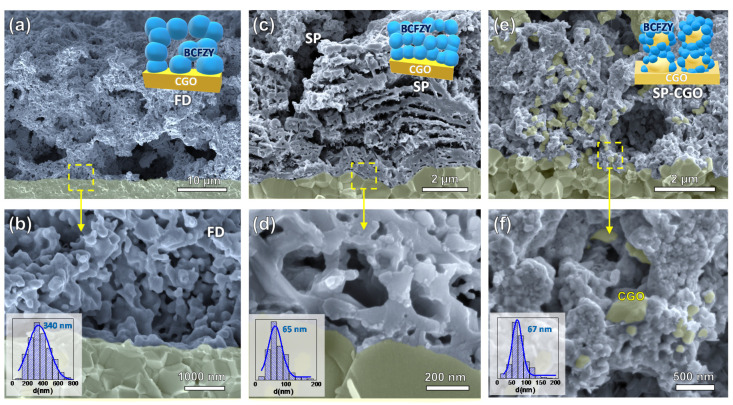
Scanning electron microscopy (SEM) of the different electrode architectures obtained by (**a**,**b**) freeze-drying and spray-pyrolysis deposition onto (**c**,**d**) polished CGO and (**e**,**f**) porous CGO backbone. The grain size distributions are included in the insets.

SEM cross-section images of the three electrode architectures at different magnifications are shown in Figure 2. The BCFZY electrodes prepared by FD and deposited by the traditional screen-printing technique have a thickness of ~25 µm (Figure 2a), presenting a high porosity and constituted by aggregates of particles of approximately 340 nm diameter (Figure 2b). 

Electrodes prepared by SP onto polished CGO pellets show much lower thickness, about ~7 µm (Figure 2c). A laminated microstructure is clearly observed due to the multiple spray coating steps and the decomposition of the organic residues after the post-deposition thermal treatment. Such morphologies, typical of a spinodal phase decomposition, have been previously observed in related materials and they are associated with the presence of EDTA in the precursor solution [41]. The SP electrode is highly porous due to the low deposition temperature and the presence of residual organic materials, creating a porous microstructure after the final heat treatment in the furnace. Figure 2d, at higher magnification, shows that the electrode is formed by well sintered particles and strong adherence to the electrolyte, ensuring high mechanical strength and low contact ohmic resistance. The average grain size of 65 nm is much lower than those obtained by the traditional FD method. 

BCFZY cathodes deposited by spray-pyrolysis onto a porous CGO backbone have a quite different morphology as can be observed in Figure 2e. The large grains with a diameter of approximately 400 nm correspond to the CGO backbone and the smaller ones are ascribed to BCFZY. Higher magnification SEM images clearly reveal that CGO grains are completely coated by the BCFZY nanoparticles with a size of 67 nm. Thus, this electrode morphology provides an extended triple phase boundary length for the ORR. In addition, the CGO scaffold provides an efficient conduction path for the oxide ion beyond the electrolyte/electrode interface (Figure 2f). It is worth noting that the low deposition temperature and low flow rate used allow the infiltration of the partially decomposed BCFZY precursors into the CGO backbone during the deposition process. This results in a high mass load of BCFZY, ~25 wt. %, in only one step, in contrast to the classical wet infiltration method, where up to 10 impregnation/calcination steps are need to obtain the same active phase loading [42]. Moreover, this method is potentially scalable from lab scale research to industrial scale production, unlike the classical wet infiltration method.

### 3.3. Electrochemical Properties

**Figure 3:**
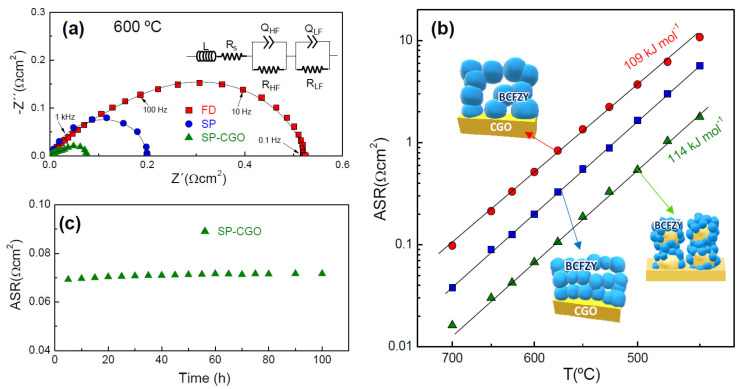
(**a**) Impedance spectra of the symmetrical cells and the equivalent circuit used to simulate the data. (**b**) Polarization resistance as a function of the temperature. (**c**) ASR over time at 600 °C.

Figure 3a shows the impedance spectra for the different electrode architectures at 600 °C in air. Notice that the ohmic resistance was subtracted for better comparison of the electrode polarization response. All the spectra show similar features with two different electrode contributions. Consequently, the spectra were fitted using the equivalent circuit of the inset Figure 3a, where *L* is an inductor due to the electrochemical setup and Rs a serial resistance associated with the total ohmic losses of the symmetrical cells. Since at least two contributions are discernible in the spectra, two (RQ) elements at high frequency (HF) and low frequency (LF) were used to simulate the electrode response. 

Figure 3b compares the area specific resistance (ASR) of the electrodes as a function of the temperature. In the whole temperature range studied, ASR decreases in the order: FD > SP > SP-CGO. All plots are parallel with similar values of activation energy 109−114 kJ·mol^−1^, which is practically identical to that reported for BSCF, 116 kJ·mol^−1^ [21,43]. However, the values of activation energies reported in the literature vary in a broad range depending on the preparation method, and electrolyte and gas atmosphere. For instance, Duan et al. reported the lowest value ~79.2 kJ·mol^−1^ with a CGO20 electrolyte [21]. These values change significantly in dry and wet air for BCFZY in contact with proton-conducting electrolytes, i.e., 111.6 and 80.8 kJ·mol^−1^, respectively, associated with the presence of proton conductivity in this cathode [19].

Regarding the values of ASR, these decrease from 0.52 Ω·cm^2^ for FD to 0.067 Ω·cm^2^ for SP-CGO at 600 °C. Thus, ASR is improved by a factor of 8 by microstructural engineering (Table 1). It is worth noting that these values are lower than those firstly reported by Duan et al. 0.25 Ω·cm^2^, although the differences are reduced at low temperatures, i.e., 0.54 Ω·cm^2^ for SP-CGO and 1.1 Ω·cm^2^ at 500 °C [21]. 

The ASR values of BCFZY with proton conductor electrolytes are also compared in Table 1. As can be observed, they vary significantly in dry and wet atmospheres. In this case, the results are not directly comparable with those obtained in this study because the electrochemical reaction for ORR may be different for oxide ion and proton-conducting electrolytes. Nevertheless, ASR values in the present work are lower, suggesting that the proposed microstructural designs may be used to improve the performance of proton-conducting SOFCs.

The ASR of SP-CGO remains stable over time, indicating a negligible degradation at least at low operating temperature (Figure 3c).

### Contributions of the Electrode Polarization

The overall ORR: $\frac{1}{2}O_{2}+2e^{-}\to 2O^{2-}$ is a complex multistep process, which involves gas diffusion, surface adsorption/dissociation and charge transfer reactions [5,6]. Additional reactions take place for mixed proton conductors such as the formation and desorption of H_2_O and proton migration from electrolyte to TPB. Each of these processes have a different oxygen (*pO*_2_) and water partial pressure (*pH_2_O*) dependence. Thus, the analysis of the high (R_HF_) and low (R_LF_) frequency resistance contributions on the *pO*_2_ and *pH_2_O* dependence is a useful method to identify the rate limiting steps for ORR, according to [37,38,47]:

$$R_{i}\propto {\left(pO_{2}\right)}^{-n}{\left(pH_{2}O\right)}^{-m}$$

If the water partial pressure is constant the following relation is assumed:

$$R_{i}\propto {\left(pO_{2}\right)}^{-n}$$

where *n* provides information about the type of the species involved in ORRs. 

**Figure 4:**
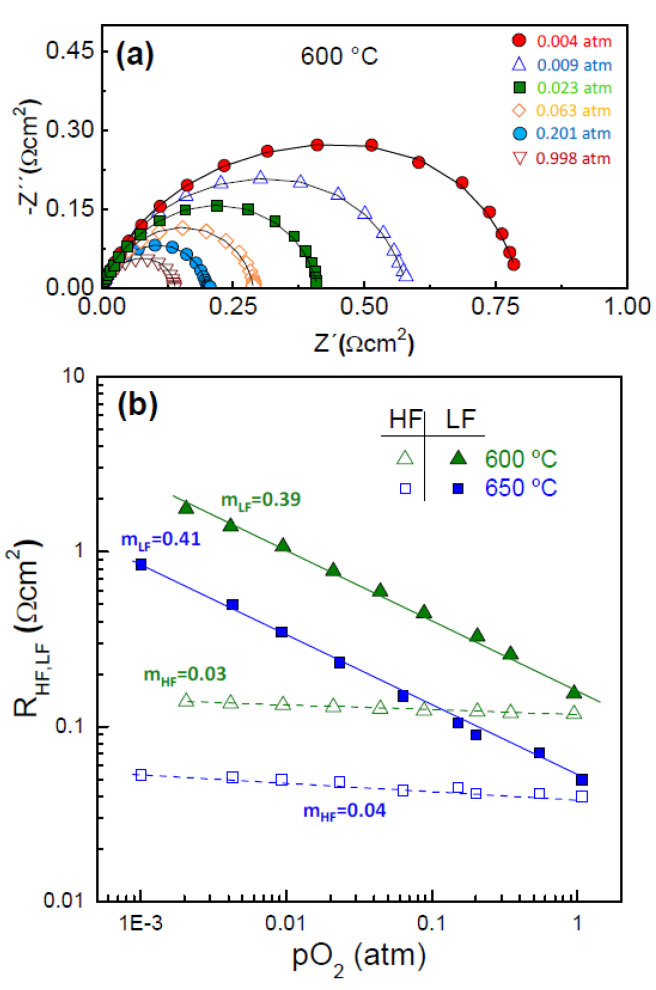
(**a**) Impedance spectra of SP electrode at 600 °C at different *pO*_2_ values. (**b**) Polarization resistance of the different electrode contributions as a function of *pO*_2_.

Figure 4a displays the impedance spectra of the SP electrode at 600 °C and various *pO*_2_ values. All the spectra are composed by two different processes and fitted by using the equivalent circuit of Figure 3a. The variation of the R_HF_ and R_LF_ resistance contributions against the *pO*_2_ is shown in Figure 4b. The HF contribution is nearly *pO*_2_-independent, suggesting that atomic or molecular oxygen are not involved in this process, and therefore, this is associated with the oxygen ion incorporation from the TPB to the electrolyte, according to: $O_{\mathrm{TPB}}^{2-}+V_{O}^{¨}\to O_{O}^{x}$. The LF contribution, with *n* close to 3/8, a high capacitance of 0.1 F·cm^−2^ and a relaxation frequency in the range of 0.1–10 Hz, is assigned to different elementary reactions in the electrode surface, i.e., atomic oxygen dissociative adsorption and diffusion of atomic oxygen, followed by a charge transfer: $O_{\mathrm{ad}}+e^{-}\to O^{-}$ [48,49]. 

**Figure 5:**
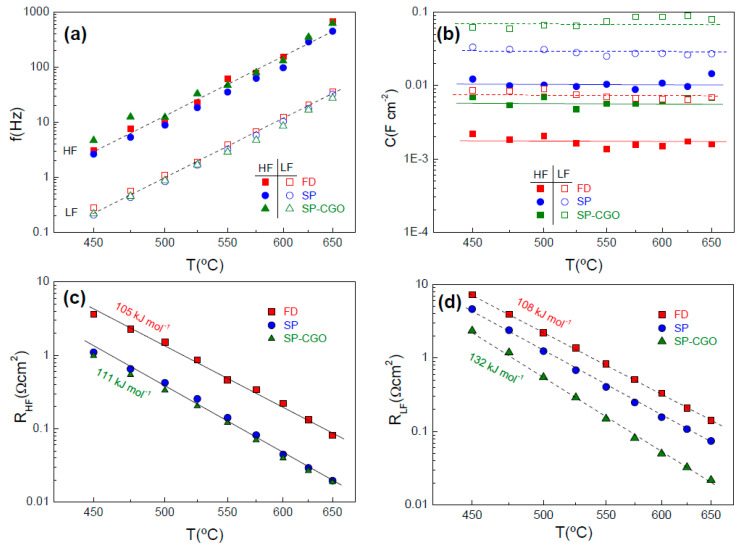
(**a**) Relaxation frequency, (**b**) capacitance and (**c**) R_HF_ and (**d**) R_LF_ resistance contributions for the different electrodes architectures.

The contributions of the electrode polarization were studied separately for a better understanding of the influence of the electrode architecture on the ORR activity. The relaxation frequency of the HF and LF processes present an Arrhenius type behaviour with the temperature with similar values for the different electrode architectures, suggesting that the same processes are involved in the ORR (Figure 5a). The LF contribution appears in the frequency range of 0.1–10 Hz, while the HF process is located at 1–1000 Hz. 

The values of capacitance are almost independent on the temperature in the range of 450–650 °C; however, they vary significantly for the different electrodes designs (Figure 5b). For both HF and LF processes, the capacitance increases in the order FD < SP < SP-CGO, which is explained by an increase of the TPB length in agreement with the particle size decrease [50]. The HF contribution, assigned to charge transfer at the BCFZY/CGO interface, has a capacitance between 1.7 mF·cm^−2^ for FD and 10 mF·cm^−2^ for SP. The LF process, attributed to reactions in the electrode surface, exhibit a large capacitance of 8 and 70 mF·cm^−2^ for FD and SP-CGO, respectively.

The Arrhenius representation of the HF and LF resistance contributions are shown in Figure 5c,d. The resistance of the HF process is much lower for those electrodes prepared by spray-pyrolysis deposition. This behaviour could be explained by the smaller particle size of the SP electrodes and reduced fabrication temperature, improving the charger transfer at the BCFZY/CGO interface, and minimizing the cation interdiffusion between the materials layers.

In contrast, the R_LF_ decreases in the following order: FD < SP < SP-CGO, which is clearly attributed to the higher electrode surface and extended TPB length for the electrochemical reactions. The corresponding activation energies are 105–111 kJ·mol^−1^ for the HF process and somewhat higher 108–132 kJ·mol^−1^ for LF process (Figure 5c,d). 

### 3.4. Fuel Cell Performance

**Figure 6:**
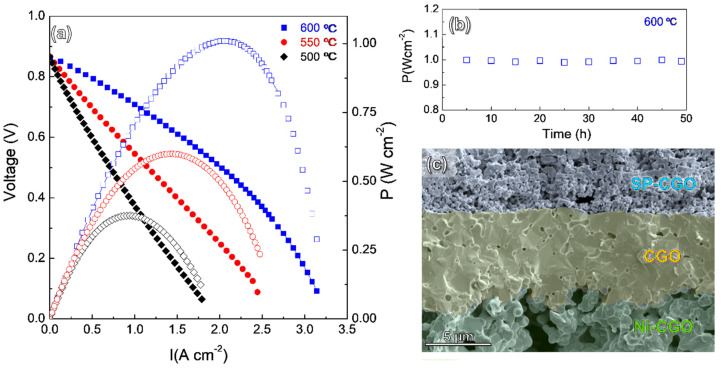
(**a**) I–V and the corresponding power density curves of the Ni-CGO supported cell with BCFZY cathode at different temperature. (**b**) Power density over time at 600 °C. (**c**) Cross-sectional SEM image of the cell after electrochemical test.

Figure 6a shows the I–V and power density curves of the cells with configuration Ni-CGO/CGO/SP-CGO. The open circuit voltage (OCV) is clearly lower than the theoretical Nernst potential (1.1 V) due to a minor electronic contribution of the CGO electrolyte. However, these are comparable to those previously reported for similar cell configuration, indicating that a leakage in the cell sealing is ruled out [21].

The maximum power densities of the cell are 1.0, 0.6 and 0.38 W·cm^−2^ at 600, 550 and 500 °C, respectively. These values are higher than those reported for BCFZY cathode in proton conductor SOFC but lower than those reported by Duan et al. 0.97 W·cm^2^ at 500 °C (Table 1). These differences are possibly explained by the different electrolyte thickness and anode microstructure because the ASR values of BCFZY, obtained in this study, are improved compared to the previous ones [19,20,21,43,44,45,46].

The cell performance was continuously tested for two days and no appreciable performance degradation was observed (Figure 6b). After that the microstructure was examined by SEM, the electrolyte has a thickness of approximately 10 µm and low porosity (Figure 6c). The electrodes are still adequately adhered to the electrolyte without visible formation of cracks or delaminations. It has to be noticed that BCFZY exhibits a thermal expansion coefficient of 21.6 × 10^−6^ K^−1^, higher than that of the CGO electrolyte ~12.5 × 10^−6^ K^−1^ [21], which might leads to mechanical instability after successive thermal cycles. The SP-CGO electrode is formed by a rigid CGO backbone coated with BCFZY particles, and therefore, the physical compatibility issues due to thermal expansion mismatch with the electrolyte are reduced. 

In summary, the proposed electrode designs allow to improve the electrochemical performance of BCFZY. In addition, the physical and the chemical compatibility issues with the electrolyte are minimized because the temperature and fabrication steps are reduced compared to traditional method. Moreover, these approaches are economic and suitable for mass production.

## 4. Conclusions

A spray-deposition method was used to obtain a BCFZY cathode with different microstructural architectures. A BCFZY cathode deposited onto polished CGO electrolyte showed a laminated-type microstructure with high porosity, particle size of 30–90 nm and good adherence to the electrolyte. The cathode prepared by spraying onto a porous CGO backbone is formed by CGO grains coated with BCFZY nanoparticles of 30–80 nm. The reduction in the particle size resulted in extended TPB sites for the electrochemical reactions and lower values of area specific resistance, i.e., 0.52, 0.2 and 0.067 Ω·cm^2^ at 600 °C for FD, SP and SP-CGO, respectively. The ASR values have been drastically reduced compared to those previously reported in the literature.

The deconvolution of the impedance spectra by equivalent circuits demonstrated that the same processes are presented, regardless of the electrode architecture. The high frequency process, attributed to charge transfer at the electrode/electrolyte interface, was improved for samples prepared by spray-pyrolysis due to the lower fabrication temperature. The low frequency process, assigned to the electrode surface, decreased in the order FD > SP > SP-CGO, confirming a clear relationship between the TPB length and resistance.

A SOFC with the optimized SP-CGO cathode demonstrated a power density of 1.0 and 0.6 W·cm^−2^ at 600 and 550 °C, respectively. These values are among the highest ones reported for a SOFC with BCFZY as cathode, demonstrating the importance of the microstructural engineering in the enhancement of the SOFC performance.

## Figures and Tables

**Table 1:** Area specific resistance, activation energy and power density values reported in the literature for BaCo_0.4_Fe_0.4_Zr_0.1_Y_0.1_O_3-δ_ cathode.

Compound	Electrolyte	ASR (Ω·cm^2^)		*E*_a_ (KJ·mol^−1^)	*P* (W·cm^−2^) (600 °C)	Reference
		500 °C	600 °C			
FD	CGO	3.71	0.52	109	-	This work
SP	CGO	1.65	0.2	114	-	This work
SP-CGO	CGO	0.54	0.067	114	1.0	This work
BCFZY	CGO20	1.1 (dry)	0.25 (dry)	79.2 (dry)	0.97 (500 °C)	[21]
BCFZY	YSZ	-	0.39 (650 °C)	-	1.07 (650 °C)	[44]
BCFZY	BaZr_0.1_Ce_0.7_Y_0.2_O_3-δ_	2 (dry) 0.5 (wet)	0.2 (dry) 0.1 (wet)	111.6 (dry) 80.8 (wet)	0.67	[19]
BCFZY	BaCe_0.7_Zr_0.1_Y_0.1_Yb_0.1_O_3−δ_	2.0 (wet)	0.6 (wet)	88.8 (wet)	0.65	[20]
BCFZY	BaZr_0.2_Ce_0.7_Y_0.1_O_3_	-	0.81	105.2	0.07	[45]
BCFZY	BaZr_0.1_Ce_0.7_Y_0.2_O_3-δ_	1.45 (550 °C)	-	100.2	0.25	[46]
BSCF	CGO	0.6	0.07	116	1.01	[43]
